# Supplementary material for: Breaking Barriers: AI’s Influence on Pathology and Oncology in Resource-Scarce Medical Systems
Source: Cancers (Basel). 2023 Dec 2;15(23):5692. doi: 10.3390/cancers15235692 (PMC10705335; doi:10.3390/cancers15235692)
Supplement: Supplementary file 1 [file cancers-15-05692-s001.zip › cancers-2718979-supplementary.pdf]

| Reference | Study objective | Description | Model | Results |
|---|---|---|---|---|
| Kanavati, 2021 [13] | Tumor classification | Classify transbronchial biopsies into adenocarcinoma, squamos cell carcinoma, small cell carcinoma and non-neoplastic | EfficientNet-B1 | AUC 0.94-0.99 |
| Yang, 2021 [14] | Tumor classification | Six-category classification for main types of lung cancer and tuberculosis, organizing pneumonia and normal lung | EfficientNet-B5 | AUC 0.918-0.978 |
| Iizuka, 2020 [15] | Tumor classification | Classification of gastrointestinal biopsies into adenocarcinoma, adenoma and non-neoplastic in both stomach and colon | Inception v3 | AUC 0.96-0.99 |
| Byeon, 2022 [16] | Tumor classification | Six-class classification for main types of benign colonic polyps as well as colorectal cancer | DenseNet-161 | Accuracy 97.3% |
| Bulten, 2020 [9] | Tumor classification | Classification of prostate biopsies into benign versus malignant and Gleason grading | U-Net | AUC 0.99 for benign versus malignant |
| Bulten, 2021 [17] | Tumor classification | Agreement of pathologists when grading prostate cancer with AI | U-Net | Cohen's kappa 0.799 vs 0.872 with AI |
| Lu, 2021 [18] | Tumor classification | Predicting origins in metastases of unkown primaries | ResNet encoder and attention pooling | Top-3 agreement of 82% |
| Wu, 2023 [23] | Biomarker quantification | HER2 scoring | CNN | Accuracy 0.93 with AI vs 0.8 without AI |
| Wang, 2021 [24] | Biomarker quantification | PD-L1 scoring in breast cancer | Linknet | Concordance improved from 0.674 to 0.950 |
| Naso, 2021 [26] | Biomarker quantification | PD-L1 scoring in NSCLC | Random trees classifier | Concordance of 0.925 in automated scoring |
| Liu, 2021 [27] | Biomarker quantification | PD-L1 scoring in lung squamous cell carcinoma | Res50-UNet | MAE 0.865<br>Pearson correlation 0.9436 |
| Li, 2022 [28] | Biomarker quantification | Ki-67 scoring | Inception v3 and ResNet | Intra-group correlation coefficient 0.905 |
| Fulawka, 2022 [29] | Biomarker quantification | Ki-67 scoring | DenseNet and fuzzy-set interpretation | MAE 0.024 |
| Xu, 2018 [32] | Survival prediction | WSI risk and survival prediction for bladder, breast carcinomas and glioblastoma | Graph-based CNNs | MAEs: 123.2 for bladder, 167.5 for breast, 303.3 for glioblastoma |

| | | | | |
|---|---|---|---|---|
| Zhu, 2017 [33] | Survival prediction | Survival analysis from WSI patch clustering | CNN | C-index 0.510-0.703 |
| Vale-Silva, 2021 [34] | Survival prediction | Survival prediction using WSI, clinical data, CNV, gene and miRNA expression | ResNet-50 | Time-dependent C-indexes 0.554-0.988 |
| Coudray, 2021 [35] | Prediction of molecular alterations from H&E | Predict common mutations in NSCLC | Inception v3 | AUC 0.733-0.856 |
| Wagner, 2023 [36] | Prediction of molecular alterations from H&E | Prediction of MSI in colorectal cancer | Transformer | AUC 0.99<br>NPV 0.99 |
| Fremond, 2023 [37] | Prediction of molecular alterations from H&E | Prediction of MMR, POLE and p53 alterations in endometrial cancer | Attention-based | AUROC 0.844-0.928 |
| Chen,2020 [38] | Prediction of molecular alterations from H&E | Prediction of common mutations in HCC | Inception v3 | AUC 0.71-0.89 |
| Jiang, 2021 [39] | Prediction of molecular alterations from H&E | Prediction of IDH mutation status in low-grade gliomas | ResNet-18 | AUC 0.667 |
| Yan, 2023 [40] | Prediction of molecular alterations from H&E | Prediction of common mutations in bladder cancer | Hierarchical deep multi-instance learning | AUC > 0.83 |
| Qu, 2021 [41] | Prediction of molecular alterations from H&E | Prediction of common mutations in breast cancer | ResNet-101 | AUC 0.729-0.852 |
| Wang, 2021 [42] | Prediction of molecular alterations from H&E | Prediction of germline BRCA mutations | ResNet | AUC 0.766 |
| Bourgade, 2023 [43] | Prediction of molecular alterations from H&E | Prediction of somatic BRCA mutations | ResNet | AUC 0.681 |
| Dolezal, 2021 [44] | Prediction of molecular alterations from H&E | Prediction of BRAF-RAS score in thyroid neoplasms | Xception | AUC 0.97-0.99 |
| Kim, 2022 [45] | Prediction of molecular alterations from H&E | Prediction of BRAF mutation in melanoma | Inception v3 and pathomic features | AUC 0.89 |
| Jain, 2020 [46] | Prediction of molecular alterations from H&E | Prediction of TMB high or low status in lung adenocarcinoma | Inception v3 | AUC 0.92 |
